# Ascitic autotaxin as a potential prognostic, diagnostic, and therapeutic target for epithelial ovarian cancer

**DOI:** 10.1038/s41416-023-02355-2

**Published:** 2023-08-18

**Authors:** Jung-A Choi, Hyosun Kim, Hyunja Kwon, Elizabeth Hyeji Lee, Hanbyoul Cho, Joon-Yong Chung, Jae-Hoon Kim

**Affiliations:** 1 Department of Obstetrics and Gynecology, Gangnam Severance Hospital, Yonsei University College of Medicine, Seoul, 03722 Korea; 2 Institute of Women’s Life Medical Science, Yonsei University College of Medicine, Seoul, 03722 Republic of Korea; 3 Molecular Imaging Branch, Center for Cancer Research, National Cancer Institute, National Institutes of Health, Bethesda, MD 20892 USA

**Keywords:** Tumour biomarkers, Ovarian cancer

## Abstract

**Background:**

Malignant ascites contributes to the metastatic process by facilitating the multifocal dissemination of ovarian tumour cells onto the peritoneal surface. However, the prognostic and diagnostic relevance of ascitic fluid remains largely unknown. Herein, we investigated the potential clinical value and therapeutic utility of ascitic autotaxin (ATX) in epithelial ovarian cancer (EOC).

**Methods:**

ATX expression was assessed in clinical samples. Spheroid-forming assay, real-time PCR, western blot analysis, invadopodia assay, and adhesion assays were performed.

**Results:**

Ascitic ATX expression was highly elevated in patients with ovarian cancer compared to those with benign ascites and was associated with advanced stage, high grade, and a short disease-free period in patients with EOC. Combining the diagnostic ability of ascitic ATX and serum CA-125 levels significantly improved the area under the curve (AUC) value for EOC compared to serum CA125 level alone. This marker combination showed a large odds ratio for short disease-free period in high-risk EOC groups. Functional studies revealed that ascitic ATX was required for maintaining cancer stem cell-like characteristics and invadopodia formation.

**Conclusion:**

Ascitic ATX levels may serve as a useful prognostic indicator for predicting aggressive behaviour in EOC. ATX-linked invadopodia are a potential target to prevent peritoneal dissemination in ovarian cancer.

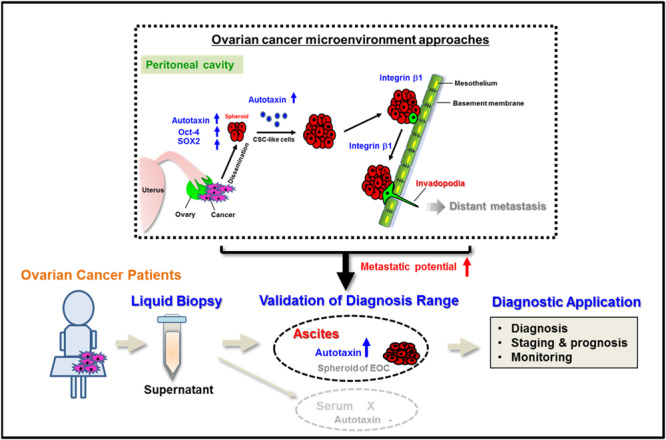

## Background

Owing to the absence of effective screening tests for early diagnosis, ovarian cancer is generally detected only after it has reached an advanced stage. The lack of early detection methods is closely related to high disease recurrence and low survival rate (less than 40%) of patients with ovarian cancer [1]. Although serum CA125 is presently considered the most representative biomarker for ovarian cancer, its accuracy is limited by low sensitivity and specificity [2]. Therefore, a reliable diagnosis strategy is urgently needed to predict the risk of ovarian cancer development at an early stage.

Liquid biopsy-derived biomarkers for ovarian cancer have been identified and are under investigation [3, 4]. Blood is typically used as a liquid biopsy sample for cancer diagnosis and screening [3, 4]. However, it may not accurately reflect the heterogeneity and microenvironment of a tumour and is also associated with low sensitivity and specificity. Growing evidence suggests that malignant ascitic fluid is a common feature in patients with ovarian cancer [5, 6], serving as an important tumour microenvironment wherein the hallmarks of ovarian cancer are fostered [5–7]. Notably, tumour cells in the ascites are present as spheroids, which are associated with the molecular phenotype of chemoresistant ovarian tumours and mimic the traits of cancer stem cell (CSC)-like cells [8, 9]. Metastatic spheroids are generated via multicellular detachment during intra-abdominal dissemination of epithelial ovarian cancer (EOC) cells [9]. These spheroids adhere to the mesothelial extracellular matrix (ECM), which allows them to anchor onto the pelvic organs as secondary lesions [10, 11]. These metastatic processes may be further accelerated by tumour-promoting factors, such as hypoxia-inducible factor, tumour necrosis factor, endothelin 1, interleukin-1β, matrix metalloproteinases, insulin-like growth factor 1, epidermal growth factor, and transforming growth factor β [5, 6]. These studies suggest that elements within malignant ascites are closely related to the diagnosis as well as prognosis of cancer and could be useful in screening high-risk patients for recurrence. However, a diagnosis and treatment platform using ascitic samples has not been established yet.

Autotaxin (ATX) is a member of the ectonucleotide pyrophosphate and phosphatase (ENPP) family and primarily catalyses the hydrolysis of lysophosphatidylcholine, resulting in the production of lysophosphatidic acid (LPA) [12]. Compelling evidence suggests that the biological activities of the ATX-LPA signalling axis regulate the progression, angiogenesis, and metastasis of tumours in various types of cancer [13–25]. ATX expression is elevated in neuroblastoma [13], glioblastoma [14], hepatocarcinoma [15], B-cell lymphoma [16], melanoma [17], breast cancer [18], and ovarian cancer [19]. An association between ATX expression and the invasiveness of cancer cells has been reported in breast cancer [18] and glioblastoma multiforme [14]. Persistently high ATX levels are reported to be associated with tumorigenesis in animal models of breast cancer, melanoma, and neuroblastoma [17, 18, 21]. Moreover, ATX expression has been reported to be elevated in poorly differentiated tumours and is correlated with the invasiveness of cancer cells [22–24]. LPA induces a pro-metastatic transcriptional signature in ovarian cancer cells that enables the prediction of poor clinical outcomes [25], suggesting a higher metastatic potential in ATX-expressing tumours. However, the value of ATX as a diagnostic and prognostic marker in patients with cancer remains controversial. Nakamura et al. suggested that serum ATX levels are unsuitable as a marker for prostate cancer, as assessed from the results of lysophospholipase D activity [26]. Similarly, patients with ovarian cancer do not show elevated serum ATX levels compared with healthy subjects [27]. Another group has reported no difference between the plasma LPA level in patients with ovarian cancer and that in healthy controls [28]. Accordingly, it is necessary to re-evaluate the clinical value of ATX by applying a different diagnostic approach. No reports have clarified the clinical significance of ascitic ATX measurement in ovarian cancer.

Here, we compared the relevance of ascite fluids for ovarian cancer prognosis and diagnosis. We developed a method for predicting poor prognosis and disease stage based on validation and expression patterns of ascites ATX in ovarian cancer. In addition, we investigated the functional role of ascitic ATX in the aggressive behaviour of ovarian cancer.

## Materials and methods

Details are described in the supplementary methods.

## Results

### Differential expression of ATX according to liquid biopsy-type in patients with EOC

**Fig. 1:**
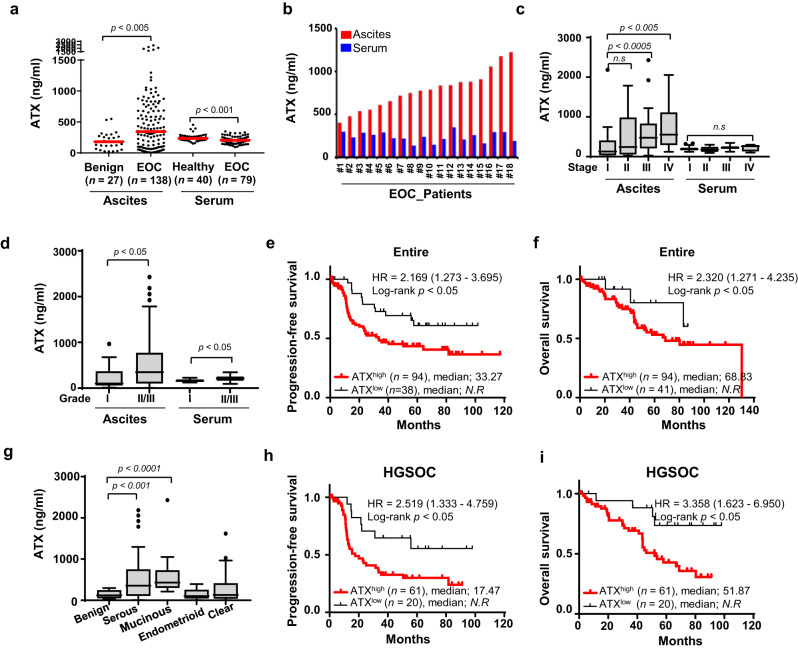
Increased autotaxin (ATX) levels in ascites but not serum indicate poor prognosis in patients with epithelial ovarian cancer (EOC). **a** ATX levels in serum and ascitic samples from patients with EOC compared to those in the healthy controls and patients with benign tumours. ATX concentration was measured using ELISA. *n* = number of patients. **b** Comparison of ATX levels in serum and ascites in matched patients with EOC. ATX concentration was measured using ELISA. **c**, **d** Box and whiskers plots showing ATX expression according to FIGO stage (**c**) and grade (**d**) in the ascites and serum samples of patients with EOC. The Mann–Whitney *U* test was used to evaluate statistical significance. **e**, **f** Kaplan–Meier curves used to evaluate progression-free survival (PFS) (**e**) and overall survival (OS) (**f**) in patients with entire ovarian cancer. Patients were classified into high (>30th percentile) and low (<30th percentile) ATX expression groups according to the 30th percentile of ATX expression. HR hazard ratio, 95% CI 95% confidence interval, N.R. not reached, *n* number of patients. **g** Box and whiskers plots showing ATX expression in the ascites from patients with different histological subtypes of EOC. The Mann–Whitney *U* test was used to evaluate statistical significance. **h**, **i** Kaplan–Meier curves to assess PFS and OS in patients with HGSOC tumours. Patients were classified into high (>30th percentile) and low (<30th percentile) ATX expression groups according to the 30th percentile of ATX expression. HR hazard ratio, 95% CI 95% confidence interval, N.R. not reached, *n* number of patients.

To elucidate the clinical significance of ATX expression in patients with EOC, the expression level of ATX from liquid biopsies was analysed in the ascites in patients with benign tumours (*n* = 27) and EOC (*n* = 138) and the serum samples from healthy individuals (*n* = 40) and patients with EOC (*n* = 79) using ELISA. The detailed clinicopathological characteristics are shown in Supplementary Table S1. Box plot analysis indicated that ATX expression was strongly enhanced in ascites derived from patients with EOC (470.7 ± 461.7 ng/ml) compared to that in ascites from patients with benign tumours (191.2 ± 141.1 ng/ml) (Fig. 1a). However, ATX levels were lower (206.2 ± 60.0 ng/ml) in the sera of patients with EOC compared to those of healthy individuals (248.6 ± 62.8 ng/ml) (Fig. 1a). Next, we evaluated the effect of the enhanced ascitic ATX levels on serum ATX levels in paired patients with EOC. Analysis of samples from 26 patients with EOC showed that the increased ATX levels in the ascites did not influence the serum ATX content (Fig. 1b). These results indicate that higher levels of ATX are present within the ascites than in the sera of patients with EOC.

### High ascitic ATX levels are associated with a poor outcome in ovarian cancer

We assessed ATX levels in patients with various clinicopathological characteristics. The expression levels of ascitic ATX were associated with advanced-stage (Stage III/IV) and high-grade (Grade II/III) cancers (Fig. 1c, d), whereas no significant association was observed between serum ATX level and the grade or stage of EOC (Fig. 1c, d). These results indicate that higher levels of ascitic ATX correlated with high-risk ovarian cancer. Next, we examined the relationship between ascitic ATX expression and patient survival. The patients were categorized into high-expression and low-expression groups based on their 30th percentile cut-offs (130.9 ng/ml), which were within the range of ascitic ATX levels in patients with benign tumours. The detailed clinicopathological characteristics of the patients with EOC are shown in Supplementary Table S1. The Kaplan–Meier plots revealed that EOC patients with high ascitic ATX levels exhibited a shorter progression-free survival (PFS) period (median; 33.27 months) than those with low ATX levels (median; *not reached*), (Fig. 1e; Hazard ratio [HR] = 2.169, *P* < 0.05). A similar significant association was observed between overall survival (OS) and high ascitic ATX levels (Fig. 1f). These results indicate that patients with high ascitic ATX levels exhibit poor PFS and OS prognoses.

### Short PFS of high-grade serous carcinoma is correlated with higher ascitic ATX expression

Given that high-grade serous ovarian carcinoma (HGSOC) is the most common and deadliest type of ovarian cancer [1], we investigated the expression of ascitic ATX along with the histological subtypes of EOC. We observed that serous EOC exhibited the strongest ATX expression among all subtypes, followed by mucinous-type EOC (Fig. 1g); however, no correlation was observed between ATX expression and endometrioid ovarian cancers. Next, we evaluated the impact of ascitic ATX on serous EOC. Ascitic ATX levels were higher in advanced-stage (Stage III/IV) cancer than those in early stages of serous-type ovarian cancer (Supplementary Fig. S1a, *P* < 0.05). Serous with Grade III showed significantly higher ascitic ATX levels than Grade I (Supplementary Fig. S1b).

Next, we analysed whether high ascitic ATX concentrations in HGSOC are correlated with the PFS and OS. The detailed clinicopathological characteristics are shown in Supplementary Table S2. Overall, the median PFS of patients with HGSOC with elevated ascitic ATX level was 17.47 months compared to *not reached* in those with low ATX expression (Fig. 1h; HR = 2.519, *P* < 0.01). The median OS in patients with HGSOC with elevated ascitic ATX level was 51.87 months compared to *not reached* in those with low ATX expression (Fig. 1i; HR = 3.358, *P* < 0.05), indicating that ascitic ATX levels could predict clinical outcomes, specifically PFS and OS in patients with HGSOC. These results suggest that the elevated ascitic ATX levels in EOC are associated with a shorter PFS and OS in patients with high-grade serous-type tumours.

Next, we evaluated the impact of ascitic ATX levels on patient prognosis to investigate whether ascitic ATX is a superior biomarker compared to other clinical risk factors. In the entire EOC, multivariable analysis revealed that ascitic ATX levels (*P* < 0.05) were independent prognostic factors for PFS (Table 1). The Cox proportional hazards model also showed that elevated ascitic ATX levels (HR = 2.20, 95% confidence interval (CI) = 1.00–4.85, *P* < 0.05) and serous cell type (HR = 3.19, 95% CI = 1.16–8.73, *P* < 0.05), were independent prognostic factors of OS. A significant association was observed between OS and high ascitic ATX levels in HGSOC (Table 1). These results show that ascitic ATX levels are correlated with patient survival rate in ovarian cancer.

**Table 1:** Univariate and multivariable analysis of the associations between prognostic variables and disease-free and overall survival in patients with epithelial ovarian cancer.

Variable		Disease-free survival hazard ratio [95% CI], *P* value		Overall survival hazard ratio [95% CI], *P* value	
		Univariate	Multivariable	Univariate	Multivariable
Entire EOC	FIGO Stage (III/IV)	5.86, [2.48–13.82], 0.000^*^	NA	2.296, [1.10–4.76], 0.003^*^	NA
	Grade (II/III)	2.13, [0.77–5.88], 0.144	1.50, [0.52 –4.31], 0.443	1.28 [0.45 –3.62], 0.629	0.61, [0.20 –1.86], 0.386
	Histology (Serous)	2.07, [1.14 –3.77], 0.017^*^	1.55, [0.82–2.94], 0.177	4.04, [1.59–10.26], 0.003^*^	3.19, [1.16–8.73], 0.024^*^
	CA125^high^	3.84, [1.20–12.3], 0.023^*^	3.19, [0.77–13.19], 0.109	4.36, [1.05–18.07], 0.042^*^	2.13, [0.50–8.99], 0.300
	Age(≥ 50)	1.66, [0.92–2.96], 0.087	NA	1.57, [0.78–3.78], 0.205	NA
	ATX^High^	1.89, [1.04–3.43], 0.036^*^	1.95, [1.04–3.63], 0.036^*^	2.29, [1.10–4.76], 0.026^*^	2.20, [1.00–4.85], 0.049^*^
HGSOC	FIGO Stage (III/IV)	5.48, [1.31–22.88], 0.020^*^	NA	28.39, [0.35–2242.6], 0.133	NA
	CA125^High^	2.20, [0.53 –9.13], 0.275	2.19, [0.52–9.09], 0.280	1.74, [0.41–7.30], 0.444	1.44, [0.34 –6.07], 0.615
	Age (≥ 50)	1.53, [0.64–3.63], 0.329	1.71, [0.72–4.07], 0.221	0.78, [0.33–1.80], 0.560	0.90, [0.39–2.10], 0.820
	ATX^high^	2.24, [1.04–4.82], 0.039^*^	2.29, [1.06–4.95], 0.034^*^	3.38, [1.19–9.63], 0.022^*^	3.26, [1.13–9.35], 0.028^*^

*FIGO* International Federation of Gynecology and Obstetrics, *CA125* cancer antigen 125, *CI* confidence interval, *NA* not applicable.

*P < 0.05.

### Diagnostic value of ascitic ATX combined with serum CA125

**Fig. 2:**
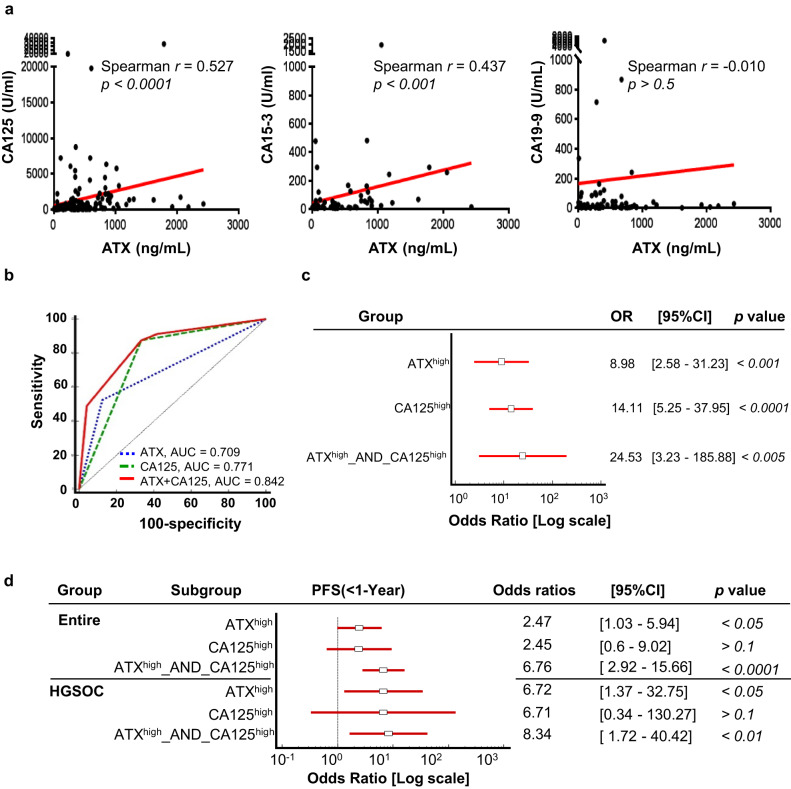
ROC curve analysis for ascitic ATX levels for the diagnosis of EOC. **a** Linear regression curve between ascitic ATX and transitional serum biomarkers in ovarian cancer. Serum levels of CA125, CA15-3, and CA19-9 were used as transitional markers in patients with ovarian cancer. Spearman’s correlation analysis revealed a significant correlation between ascitic ATX and serum CA125 levels. **b** ROC curves for ascitic ATX and serum CA125 in EOC. **c** Forest plot shows the odds ratios with 95% confidence intervals for the association between patients with EOC and either ascitic ATX or serum CA125. **d** Forest plot shows the odds ratios with 95% confidence intervals for the association between patients with EOC and either ascitic ATX or serum CA125 with an outcome of 1-year PFS.

To investigate the diagnostic value of ascitic ATX in EOC, we first examined the correlation between ascitic ATX expression and the serum levels of CA125, CA15-3, and CA19-9, the known clinical diagnostic biomarkers for ovarian cancer [2]. Spearman’s correlation analysis revealed that ascitic ATX expression had a strong positive correlation with the serum levels of CA125 (Spearman *r* = 0.527, *P* < 0.0001) and CA15-3 (Spearman *r* = 0.437, *P* < 0.0005), but not with those of CA19-9 (Spearman *r* = −0.01, *P* > 0.5) (Fig. 2a). Therefore, we compared the utility of ascitic ATX and serum CA125 as diagnostic tools for ovarian cancer using an ROC curve analysis. The optimal cut-off value of 332.8 ng/ml for ascitic ATX level was determined from the distribution of values at the highest Youden index for the diagnosis of ovarian cancer, with a sensitivity of 52.90% and a specificity of 88.89% (Supplementary Table S3). The area under the curve (AUC) value for distinguishing patients with EOC from those with benign tumours was 0.709 for ascitic ATX [95% CI = 0.633–0.777] and 0.771 for serum CA125 [95% CI = 0.699–0.834]. Additionally, ascitic ATX had higher specificity (88.89%) and positive predictive value (PPV; 96.1%) compared to serum CA125 (Supplementary Table S3; 66.67% and 93.7%, respectively).

Next, we elucidated the diagnostic relevance of ascitic ATX and serum CA125 as a combined ovarian cancer diagnostic marker. The combination of ascitic ATX with serum CA125 increased the AUC value to 0.842, which was higher than the value for serum CA125 alone (AUC = 0.771) (Fig. 2b and Supplementary Table S3). These results suggest that ascitic ATX, used in conjunction with CA125, is a potential diagnostic and screening biomarker for ovarian cancer with a high diagnostic prediction rate. We further assessed the incidence risk of ovarian cancer by comparing the odds ratios in the following three subgroups: ATX^high^ (ascitic ATX ≥ 332.8 ng/ml), CA125^high^ (serum CA125 ≥ 35 U/ml), and ATX^high^_AND_CA125^high^ (ascitic ATX ≥ 332.8 ng/ml and serum CA125 ≥ 35 U/ml). As shown in Fig. 2c, the forest plot showed that the odds ratio for EOC was 8.98 (*P* < 0.001) for ATX^high^ and 14.11 (*P* < 0.0001) for CA125^high^. Remarkably, when these markers were combined, the odds ratio increased to 24.53 for ATX^high^_AND_CA125^high^ (*P* < 0.005). Finally, we evaluated the incidence rate of PFS of less than 1 year when used with serum CA125, compared to ascitic ATX alone (Fig. 2d). The forest plot revealed that the odds ratio for 1-year PFS was 2.47 (*P* < 0.05) for ATX^high^ and 2.45 (*P* > 0.1) for CA125^high^. However, when these markers were combined, the odds ratio increased to 6.76 for ATX^high^_AND_CA125^high^ (*P* < 0.0001). These results suggest that diagnosis using two markers simultaneously is more likely to diagnose HGSOC. These data demonstrate that an assessment performed by combining ascitic ATX and serum CA125 levels enhances the accuracy of EOC diagnosis.

### ATX plays a critical role in maintaining the CSC-like characteristics in EOC

**Fig. 3:**
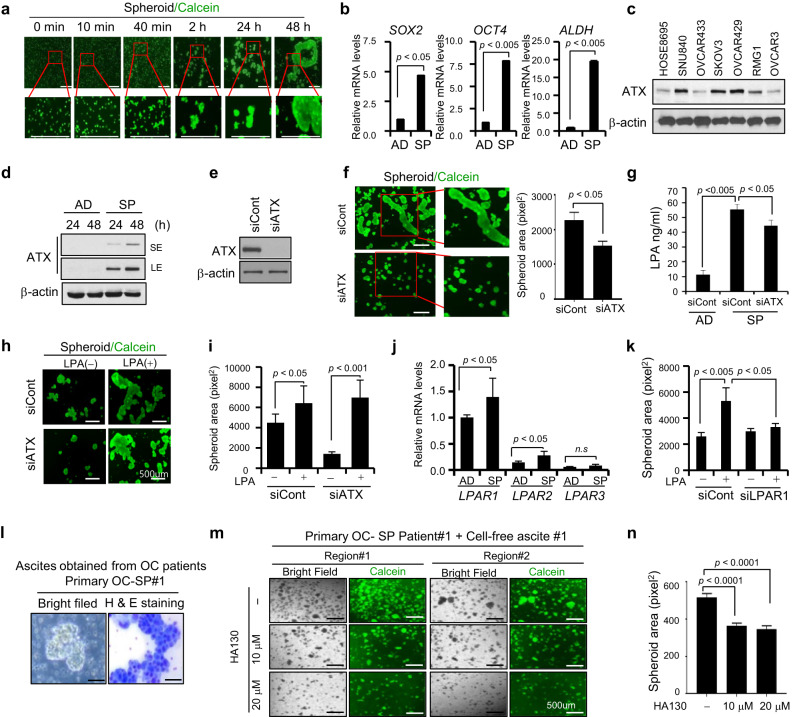
ATX is associated with maintenance of CSC-like characteristics in ovarian cancer cells. **a** Spheroid-forming ability of SKOV3 cells under non-adherent conditions. SKOV3 cells were plated onto poly-HEMA-coated dishes for 10 min to 48 h. The cells were then stained with calcein-AM for visualization and photographed using an EVOS microscope at the indicated times. Scale bars = 500 μm. **b** Increased expression of CSC-like genes in a 3D-spheroid culture of SKOV3 cells, as determined using real-time PCR. SKOV3 cells were plated on uncoated and poly-HEMA-coated dishes. The cells were subjected to real-time PCR with the specific primers for *SOX2, OCT4*, and *ALDH*. The 18 S rRNA gene was used as the endogenous control to normalize the data. **c** Immunoblotting analysis of ATX expression in ovarian cancer cell lines (SNU840, OVCAR433, SKOV3, OVCAR429, and OVCAR3) and immortalized HOSE cells (IHOSE8695). Cell lysates were subjected to immunoblotting with an anti-ATX antibody. Anti-β-actin was used as the loading control. **d** Immunoblotting analysis of ATX expression in suspended SKOV3 cells. Cells were plated onto poly-HEMA-coated or uncoated dishes for 24 and 48 h. Cell lysates were subjected to immunoblotting with an anti-ATX antibody. Anti-β-actin was used as the loading control. AD adhesion, SP suspension. **e** Representative western blot analysis of ATX expression in SKOV3 cells transfected with siATX or siControl. **f** Effect of ATX depletion on spheroid formation in SKOV3 cells. Cells were transfected with *ATX*-specific siRNA. After 24 h, the cells were plated in poly-HEMA-coated dishes, incubated for 24 h, stained with calcein, and photographed with EVOS (left). Spheroid areas were calculated using the ImageJ software (right). Scale bar = 100 μm. **g** LPA levels were determined using ELISA in the indicated media for SKOV3 cells. AD adhesion, SP suspension. **h**, **i** Effect of low ATX level on LPA-induced spheroid formation in SKOV3 cells. Cells with ATX siRNA were plated in poly-HEMA-coated dishes with LPA (1 μM) for 24 h. Then, spheroid cells were stained with calcein (left). Spheroid areas were calculated using ImageJ (right). **j** Expression of LPAR genes in the 3D-spheroid culture of SKOV3 cells, as determined using real-time PCR. The cells were subjected to real-time PCR with the specific primers for *LPAR1*, *LPAR2*, and *LPAR3*. **k** Effect of LPA on LPAR1-silencing SKOV3 cells. Cells with siRNA LPAR1 were incubated in poly-HEMA-coated dishes for 24 h. Then, cells were stained with calcein. Spheroid areas were calculated using ImageJ. **l** Bright-field microscopy (left) and H&E staining (right) show primary EOC spheroids isolated from the ascites derived from patients with EOC. **m**, **n** Effect of ATX inhibitor on primary ovarian cancer cells isolated from ovarian cancer patient-derived ascites. Ovarian cancer-enriched ascites were plated onto poly-HEMA-coated dishes for 24 h and were cultured with cell-free ascitic fluids in the presence or absence of 10 and 20 µM of HA130. Cells were stained with calcein-AM (**m**). Spheroid areas were calculated with ImageJ (**n**). Scale bar =  100 μm. Statistical comparisons were performed using Student’s *t* test.

Ovarian cancer cells in ascites are spheroids with CSC-like characteristics [11, 29, 30]. Therefore, we investigated the biological function of ATX in ascites by testing its ability to induce spheroid formation in 3D culture. SKOV3 cells were loaded into a poly-HEMA-coated dish and screened. Several large cell clumps and irregular 3D-spheroid shapes were observed over time (Fig. 3a). Cells with 3D-spheroid shapes showed higher expression levels of the stem cell markers *SOX2*, *OCT4*, and *ALDH* than adhered cells (Fig. 3b). Next, we examined the role of ATX in the formation of multicellular spheroids in EOC. ATX expression was elevated in various ovarian cancer cell lines, including SNU840, OVCAR433, SKOV3, OVCAR429, RMG, and OVCAR3, compared to that in the immortalized ovarian cells IHOSE8695 (Fig. 3c). An immunoblotting assay showed that ATX expression was enhanced under 3D-spheroid conditions (Fig. 3d). Silencing ATX expression markedly inhibited multicellular spheroid formation as well as LPA production (Fig. 3e–g). The reduced spheroid formation caused by ATX loss was recovered by LPA treatment (Fig. 3h, i).

We determined the role of LPA receptors in the biological function of ATX by analyzing the expression patterns of LPARs (LPAR1, LPAR2, and LPAR3) in 3D-spheroid cells. Among the three LPA receptors, LPAR1 expression was strongly enhanced in ovarian cancer cells and significantly higher in 3D spheroids than that in adhered cells (Fig. 3j and Supplementary Figs. S2 and S3). In contrast, only low expression of *LPAR2* mRNA and no significant expression of *LPAR3* mRNA were detected. Silencing LPAR1 impaired spheroid formation in ovarian cancer cells (Fig. 3k and Supplementary Figs. S2 and S3). These observations suggest that ATX-induced CSC-like characteristics are mediated by LPA and its receptor, LPAR1.

Next, we further confirmed the effects of ATX signalling on spheroid formation by ex vivo analysis using ascites derived from patients with ovarian cancer. Samples of primary EOC spheroids of various shapes and sizes from patient-derived ascites were observed using bright-field microscopy (Fig. 3l). The enriched spheroid cancer cells were loaded onto poly-HEMA-coated plates and cultured with cell-free ascitic fluids in the presence or absence of HA130, an ATX inhibitor. Treatment with HA130 impaired the formation and reduced the area of multicellular spheroids in primary EOC (Fig. 3m, n). These observations suggest that ascitic ATX is important for maintaining CSC-like characteristics in EOC.

### ATX contributes to the formation of invadopodia

**Fig. 4:**
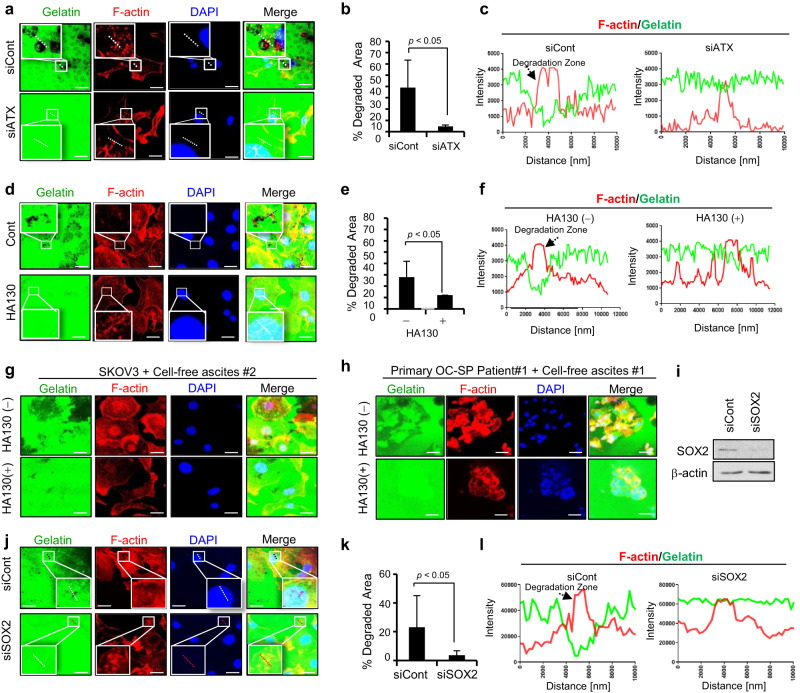
ATX is critical for the formation of invadopodia. **a**–**c** Impairment of invadopodia formation upon ATX silencing in SKOV3 cells. Cells transfected with ATX siRNA were plated onto dishes coated with FITC-labeled gelatin. After 16 h, the cells were fixed with 4% paraformaldehyde and were stained with Alexa Fluor 594-Phalloidin and DAPI. The area of degraded gelatin was visualized using confocal microscopy. The arrowhead indicates the area of FITC-gelatin degradation. The Invadopodia area was calculated using ImageJ (**b**). Representative intensity profiles of F-actin/gelatin obtained from the zoomed images. The arrowhead indicates the location of the degraded gelatin matrix in siATX-treated SKOV3 cells (**c**). **d**–**f** Effect of HA130 on invadopodia formation in SKOV3 cells. The degraded gelatin area was visualized using confocal microscopy (**d**). The invadopodia area was calculated with ImageJ (**d**). Representative intensity profiles of F-actin/gelatin obtained from the zoomed images (**f**). **g** Effect of HA130 on invadopodia formation in SKOV3 cells cultured with cell-free ascites isolated from patients with ovarian cancer. HA130-treated SKOV3 cells were cultured using cell-free ascites in a FITC-gelatin-coated dish. Scale bar = 25 µm. **h** Effect of HA130 on invadopodia formation in primary cancer cells isolated from the ascites derived from ovarian cancer patients. Primary cancer cells were obtained from the ascites of the patients and plated on a FITC-gelatin-coated dish with HA130 (10 and 20 µM) in the presence of cell-free ascites. Green; gelatin, Red; Phalloidin, Blue; DAPI. **i**–**l** Effect of *SOX2* depletion on invadopodia formation in SKOV3 cells. The cells were transfected with *SOX2* siRNA or siControl (**i**). The degraded gelatin area was visualized using confocal microscopy (**j**). The invadopodia area was calculated using the ImageJ software (**k**). Representative intensity profiles of F-actin/gelatin obtained from the zoomed image (**l**). Statistical comparisons were performed using Student’s *t* test.

Invadopodia are dynamic, actin-rich protrusive structures that can degrade the ECM and are observed in metastatic cancer cells [31]. We assessed the contribution of ATX to invadopodia formation using a FITC-labelled gelatin matrix, as multicellular spheroids formed by ovarian tumour cells indicate an increased metastatic potential [30]. Invadopodia-rich areas were observed as black spots due to FITC-gelatin matrix degradation in the accumulated F-actin filaments at the ventral surfaces of cells. Confocal microscopy images showed that silencing ATX expression with siRNA markedly suppressed invadopodia formation (Fig. 4a) in SKOV3 cells. Similar effects were observed in RMG1 and OVCAR429 cells (Supplementary Fig. S4). Quantitative analysis of the images revealed that ATX-deficient cells had >50% reduction in invadopodia formation compared with the control cells (Fig. 4b). The intensity profile of F-actin/gelatin obtained from the zoomed images showed a reduced zone upon ATX silencing (Fig. 4c). Similar results were obtained after HA130 treatment of the cells (Fig. 4d–f). Treatment with LPA recovered the impaired invadopodia formation induced by ATX silencing in SKOV3 (Supplementary Fig. S5).

To determine the clinical relevance of excess ATX present in the ascites, we evaluated the effect of ascitic fluid derived from patients with EOC on the invadopodia-forming ability of ovarian cancer cells. SKOV3 cells were cultured on a FITC-gelatin-coated plate with cell-free ascitic fluids obtained from the patients in the presence of HA130 for 24 h. Treatment with HA130 impaired invadopodia formation in SKOV3 cells (Fig. 4g), indicating that ascitic ATX is important for invadopodia formation in ovarian cancer. We further confirmed the function of ascitic ATX in invadopodia formation in a tumour microenvironment. Patient-derived, enriched cancer spheroid cells were seeded on a FITC-gelatin-coated plate with autologous ascitic fluids in the presence or absence of HA130 (Fig. 4h). Treatment with HA130 impaired invadopodia formation and reduced the invadopodia area in EOC, indicating that ascitic ATX is required for invadopodia formation in ovarian cancer. This provides direct evidence that ATX plays a critical role in invadopodia formation in EOC.

In light of our observations, to further confirm whether CSC-like characteristics are implicated in invadopodia formation, we investigated the involvement of SOX2, a potential ovarian CSC marker, in invadopodia formation. The Cancer Genome Atlas (TCGA) database analysis showed that the alteration frequency of SOX2 in ovarian cancer was the second strongest among all carcinomas (Supplementary Fig. S6). Interestingly, silencing of *SOX2* strongly reduced both the formation and the area infiltrated by invadopodia (Fig. 4i–l), indicating that CSC-like characteristics contribute to invadopodia formation in EOC.

### Integrin β1 is necessary for invadopodia formation and is a downstream target of ATX in EOC

**Fig. 5:**
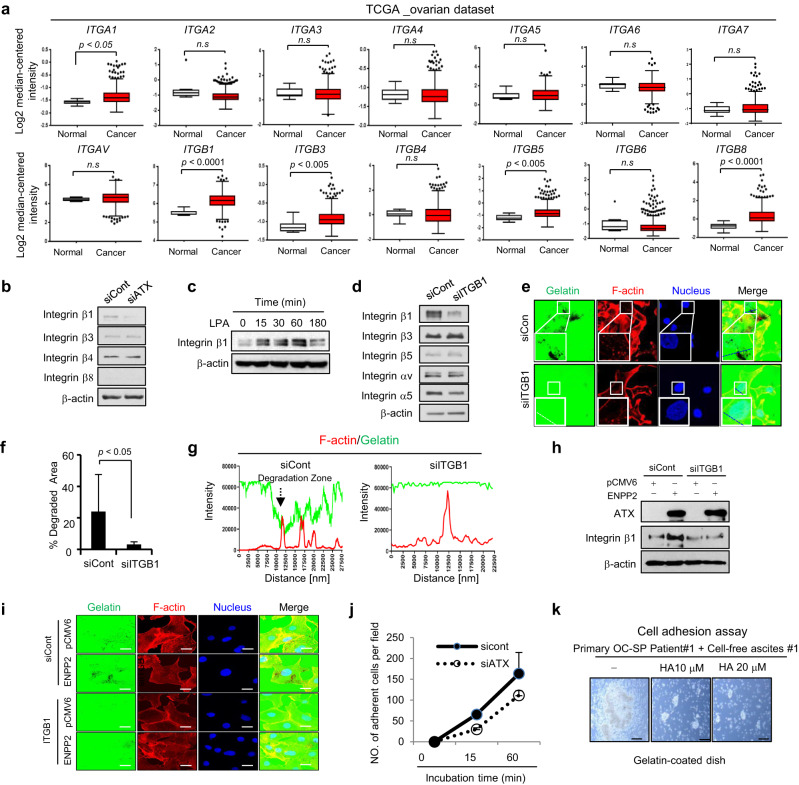
Integrin β1 is required for invadopodia formation and is a downstream target of ATX. **a** Data on the mRNA expression of the *ITGA* and *ITGB* families were obtained from the ONCOMINE database (http://www.oncomine.com). The Mann–Whitney test was used to evaluate statistical significance. **b** Impaired integrin β1 expression upon *ATX* gene silencing in SKOV3 cells. SKOV3 cells were transfected with either ATX siRNA or control siRNA. Cell lysates were subjected to immunoblotting with the indicated antibody. **c** Expression of integrin β1 in LPA-treated SKOV3 cells. Cells were treated with LPA (1 μM) for the indicated time. Cell lysates were subjected to immunoblotting with an anti-integrin β1 antibody. Anti-β-actin was used as the loading control. **d**–**f** Cells were transfected with integrin β1 siRNA. After 24 h, the knockdown of integrin β1 was confirmed using an immunoblotting assay (**d**). Invadopodia formation was visualized using confocal microscopy (**e**). The Invadopodia area was calculated using ImageJ software (**f**). Representative intensity profiles of F-actin/gelatin obtained from the zoomed images (**g**). **h**, **i** SKOV3 cells were transfected with integrin β1 siRNA in the presence or absence of PCMV6 vector expressing Myc-DDK-tagged ENPP2 (pCMV6-ENPP2) or Myc-DDK-tagged (PCMV6). After 24 h, whole cell lysates were immunoblotted with anti-ATX and anti-β-actin antibodies (**h**). For invadopodia assay, cells were cultured on coverslips coated with FITC-gelatin (green) for 16 h and then imaged using a confocal microscope (**i**). **j** ATX deficiency impairs the adhesion ability of ovarian cancer cells. SKOV3 cells stained with calcein-AM were allowed to attach to gelatin-coated six-well plates for 15 min or 60 min. Thereafter, non-adherent cells were removed, and adherent cells were visualized under a fluorescence microscope and counted. **k** Effect of HA130 on the adhesion ability of primary cancer cells isolated from the ascites derived from ovarian cancer patients. Primary ascites-derived cancer cells were plated on a gelatin-coated dish with HA130 (10 and 20 µM) in the presence of cell-free ascites and were incubated for 24 h. Adherent cells were visualized using a microscope (EVOS FL; AMG). Statistical comparisons were performed using Student’s *t* test.

As integrins are the key connection between cell-invasive protrusions and the surrounding ECM [31], we investigated whether ATX is involved in integrin signalling. We assessed the expression of integrin α and β family genes in patients with ovarian cancer using the publicly available database ONCOMINE. The mRNA levels of *ITGA1, ITGB1, ITGB3*, and *ITGB8* were found to be highly elevated in ovarian cancer tissues compared to those in normal ovarian tissues (Fig. 5a). We further focused on the functional role of the integrin β family as a downstream target of ATX in ovarian cancer progression, because it plays a more relevant role in ovarian cancer progression compared to the integrin α family [32, 33]. Immunoblotting analysis revealed that ATX depletion significantly impaired the expression of integrin β1 but not that of integrin β3 and β4 (Fig. 5b); we failed to detect integrin β8 expression. In addition, exogenous LPA increased integrin β1 expression (Fig. 5c and Supplementary Fig. S7), suggesting that integrin β1 acts downstream of ATX. Next, we investigated whether integrin β1 contributes to invadopodia formation. We confirmed specific suppression of integrin β1 expression using siRNA without affecting the expression of other integrins (Fig. 5d). Loss of integrin β1 strongly suppressed the formation and area of invadopodia in SKOV3 cells (Fig. 5e, f). Similar effects were observed in the intensity profile (Fig. 5g). Additionally, the transduction of the PCMV6-ENPP2-Myc-DDK-tagged vector expressed high levels of ATX and ITGB1 but not in the Empty vector. Furthermore, siRNA ITGB1 transfection reduced the enhanced integrin β1 expression by the enforced ATX expression in SKOV3 (Fig. 5h). The enhanced invadopodia activity by the enforced ATX expression was impaired by loss of integrin β1 (Fig. 5i). These results suggest that integrin β1 is a downstream effector of ATX and plays a dominant role in facilitating invadopodia formation.

Additionally, we monitored the effect of *ATX* depletion on the adherence ability of ovarian cancer cells. SKOV3 cells were labelled with the fluorescent dye calcein-AM prior to the adhesion assay since ascitic spheroids adhere to ECM components and mesothelial cell monolayers, which allows them to anchor to the pelvic organs as secondary lesions [9]. Enumeration of the cells that were attached to the bottom revealed that the depletion of ATX by siRNA inhibits the adherent ability of SKOV3 cells in a time-dependent manner (Fig. 5j and Supplementary Fig. S8). Moreover, the adherence of primary enriched ascitic spheroids from patients with EOC was blocked by preincubation with HA130 (10 and 20 µM) (Fig. 5k). These observations indicate a crucial role of ascitic ATX in ovarian cancer cell adhesion to ECM components.

## Discussion

Ascites cytology analysis is a standard method used to determine the presence of tumours and confirm malignant ascites. However, this method has limitations in diagnosis confirmation, prognosis and recurrence prediction, and choice in drug treatment response. EOC does not follow the traditional metastatic route through the bloodstream; instead, it spreads via the transportation of EOC cells from the primary tumour that diffuses within the peritoneal cavity [6, 7]. This difference in ovarian cancer metastasis requires consideration in developing ovarian cancer diagnosis technology. Unlike other liquid biopsies, malignant ascites have shown potential as an excellent liquid biopsy to identify the characteristics of ovarian cancer because it appears in progressive disease and is rich in metastatic substances [6–11, 34, 35]. Recent whole-exome sequencing studies analysing spheroid cells derived from ascites showed that they are lineage-independent from primary tumour cell samples in HGSOC patients, suggesting this event occurred early in tumour evolution and metastasis [36]. Even in patients with ovarian cancer with the same staging, each patient has a different response rate. Therefore, it is necessary to re-evaluate the prognostic and diagnostic values of ascites in ovarian cancer using a different diagnostic approach separate from cytology. In this study, we highlighted ATX, a potential marker of cancer stem cells and an important factor in the production of metastatic cancer infiltrators. ATX is also linked to high-risk groups, is abundant in ascites in patients with ovarian cancer, and is associated with short PFS and OS.

Accumulating evidence implicates the ATX-linked cascade in promoting tumours [13–25]. Because of this, it was proposed that the level of ATX in tumours and/or serum could constitute a biomarker for cancer aggressiveness. However, several studies have shown that serum ATX levels are not a useful biomarker for ovarian cancer [26–28]. Likewise, we observed that serum ATX levels were not elevated in patients with ovarian cancer compared to those in healthy subjects. Nevertheless, ascitic ATX levels were higher in patients with ovarian cancer than those in patients with benign tumours. This result suggests a differential profile of ATX expression in ascites and serum obtained from patients with EOC. Moreover, ascitic ATX level was higher in patients with early-stage (Stage I/II) EOC than that in those with benign tumours; ATX levels also showed significant association with advanced FIGO stages and higher grades. Low expression of ascitic ATX was associated with clinically meaningful improvements in short PFS and OS in patients with HGSOC, thus suggesting that high ascitic ATX levels could predict unfavourable outcome in patients with ovarian cancer. Although lack of information concerning residual disease at primary surgery, which is one of the stronger prognostic factors for ovarian cancer, is a major limitation of our multivariable analysis, the ascitic ATX level (*P* < 0.05) was an independent prognostic factor for PFS and OS compared to other clinical risk factors. Based on these data, we speculated that a discriminatory liquid biopsy, which directly reflects tumour characteristics, could help improve the accuracy of ovarian cancer diagnosis and prognosis and provide comprehensive information about the characteristics of heterogeneous ovarian cancer cells.

In the present study, the cut-off value of ATX (332.8 ng/ml) from our ROC analysis seemed to be able to distinguish between ovarian cancer and benign tumours. Although the AUC value of ascitic ATX (0.709) was lower than that of serum CA125 (0.771) for EOC diagnostic prediction, ascitic ATX of >333.2 ng/ml had good specificity (88.89%) and PPV (96.1%) compared to those of CA-125 as a diagnostic biomarker. This suggests that ascitic ATX testing may overcome the limitations of the low specificity of serum marker CA125 for ovarian cancer detection. Our analysis showed that the combined use of ascitic ATX and serum CA125 levels greatly improved the predictive power and accuracy of ovarian cancer diagnosis (AUC = 0.842) compared to CA125 alone (AUC = 0.771). The odds ratio analysis revealed that patients with elevated ascitic ATX and serum CA125 levels had an increased risk of moving towards the progressive stage of ovarian cancer. Thus, these results demonstrate that ascitic ATX is a novel diagnostic tool and screening marker to distinguish ovarian cancer from a benign pelvic mass and can enhance the accuracy of methods used to predict high-risk ovarian cancer. The combination of ascitic ATX and serum CA125 levels showed a higher odds ratio for 1-year PFS in HGSOC than the individual markers, indicating that ATX may also serve as a poor prognostic marker for HGSOC. Given that malignant ascites are rich in metastatic material and mainly occur at an advanced stage, it appears more suitable for predicting and monitoring high-risk patients and a poor prognosis rather than as a predictor of a cancer diagnosis.

Ascitic multicellular spheroids that mimic the traits of CSCs are considered bona fide metastatic units due to their attachment to the mesothelium and invasion of the ECM for the dissemination of ovarian cancer [34, 35, 37]. However, the interplay of CSC-like spheroids to successfully overcome the basement membrane barrier in different microenvironments remains unclear. In this context, we speculated that CSC-like characteristics mediate the formation and functions of invadopodia. The inhibition of ATX, a potential marker for CSCs, led to a strong inhibition of invadopodia formation in ovarian cancer cell lines and spheroids in the ascites-derived cancer cells of patients with EOC. Furthermore, silencing *SOX2*, an ovarian CSC marker, prevented invadopodia formation. These results support our speculation that CSC-like characteristics contribute to invadopodia formation in ovarian cancer. This idea is further supported by recent studies indicating that CD44 enables invasive tumour cells to produce functional invadopodia by promoting the phosphorylation of cortactin and recruiting MT1-MMP [38]. These phenomena may accelerate the interplay between enriched tumour-promoting factors in ascitic and hypoxic conditions in the centre of the spheroid to promote the formation and maturation of invadopodia by affecting cell metabolism and inducing hypoxia and low pH [31, 39]. Consistent with this notion, using high-resolution intravital multiphoton microscopy in mice, a recent study demonstrated that most hypoxic cells move slowly near blood vessels infiltrated by abundant invadopodia [40].

Abundant evidence indicates that integrin-mediated ECM adhesion contributes to invadopodia maturation [38, 41–43] and promotes the recruitment of MT1-MMP at focal contacts, which modulates the trafficking and release of vesicles in invadopodia [43]. Furthermore, ovarian cancer upregulates integrin β1 expression, which is required for invadopodia stability [44]. Elevated integrin β1 expression is correlated with advanced-stage cancer and poor patient survival [44]. Consistent with these reports, we observed strong integrin β1 expression in various ovarian cancer cell lines (data not shown) and in the ONCOMINE database. Moreover, MT1-MMP is not detected in the normal ovarian surface epithelium or benign ovarian tumours but is widely expressed in ovarian carcinomas, where its enzymatic activity is fundamental in promoting the migration and invasion of sub-mesothelial collagen matrices [45–48]. Therefore, invadopodia-related signalling seems to be an important process during ovarian cancer progression, promoting cancer/stroma interaction and metastatic behaviour. Integrins are considered promising therapeutic targets in ovarian cancer, particularly HGSOC [49], as they drive the attachment, migration, proliferation, and survival of tumour cells by activating FAK-dependent signalling [50]. We demonstrated that siRNA-mediated depletion of integrin β1 impaired invadopodia formation in ovarian cancer cells, indicating that integrin overexpression is correlated with poor prognosis of patients with ovarian cancer. We speculated that the ATX-integrin-linked cascade drives the formation of invadopodia and their interaction with mesothelial cells and that the subsequent signalling is important for regulating the aggressive progression of ovarian cancer. This speculation is further supported by a recent study suggesting that integrin-mediated interaction with ECM proteins on the mesothelial interface is essential for the adhesion of ovarian cancer spheroids at the site of invasion [51]. However, although the functions of these integrins are strongly dependent on the activation of FAK [33, 50] and its downstream signalling, including the PI3K/Akt- and Ras/MAPK-dependent pathways [41–43], no downregulation of Src, FAK, or AKT expression was observed following the loss of ATX or integrin β1 in ovarian cancer cells (Supplementary Fig. S9), which suggested that ATX-integrin signalling regulates the formation of invadopodia in a FAK-independent manner in ovarian cancer.

Although we observed integrin β1 expression regulation by ATX, the underlying mechanism for the correlation between ATX and integrin β1 expression in ovarian cancer remains unclear. A possible mechanism could be transcriptional regulation; however, integrin β1 mRNA was not inhibited by ATX silencing (data not shown). Another possible mechanism could be ubiquitin-mediated proteasomal degradation, controlling the stability and quality of intracellular proteins [52]. A recent study suggests that the deubiquitylase USP10 regulates cell-surface integrin β1 expression post-translationally [53]. We observed that *USP10* mRNA expression was decreased by ATX silencing in SKOV3 and OVCAR429 cells (Supplementary Fig. S10), suggesting that expression of integrin β1 may be controlled by the ATX-USP10 linked pathway. Additionally, Fulkerson et al. showed that the binding of ATX to integrin can generate LPA production in platelets [54]. Consistent with this report, we observed the interaction of ATX and integrin β1 in SKOV3 (Supplementary Fig. S11), but further studies are required to determine whether this interaction is necessary for integrin degradation.

In summary, we propose the significance of using liquid biopsy as a direct reflection of tumour characteristics for improving the accuracy of ovarian cancer prognosis and diagnosis. Our findings highlight that high expression levels of ascitic ATX correlate with poor prognosis of patients with ovarian cancer and that using ascitic ATX and serum CA125 levels as a combined biomarker showed better predictive diagnosis and prognosis power than serum CA125 levels alone. We further highlight the biological function of ascitic ATX in maintaining CSC-like characteristics in the tumour and the formation of invadopodia. Although it may be difficult to obtain ascites from healthy people for early diagnosis of ovarian cancer, these findings raise the possibility that ascitic ATX levels may help to distinguish relatively inactive and aggressive tumours and that they may be useful in identifying ovarian cancers with different drug responses even at the same stage, thereby aiding clinicians in making decisions and providing optimal treatment and monitoring.

### Supplementary information

Supplemental material

## Data Availability

Details are described in the supplemental information.
