# Supplementary material for: Ascitic autotaxin as a potential prognostic, diagnostic, and therapeutic target for epithelial ovarian cancer
Source: Br J Cancer. 2023 Aug 18;129(7):1184–94. doi: 10.1038/s41416-023-02355-2 (PMC10539369; doi:10.1038/s41416-023-02355-2)
Supplement: Supplementary file 1 — Supplemental material [file 41416_2023_2355_MOESM1_ESM.docx]

**SUPPLEMENTARY MATERIAL**

**Ascitic autotaxin as a potential prognostic, diagnostic, and therapeutic target for epithelial ovarian cancer**

Jung-A Choi, Hyosun Kim, Hyunja Kwon, Elizabeth Hyeji Lee, Hanbyoul Cho, Joon-Yong Chung, and Jae-Hoon Kim

**MATERIALS AND METHODS**

**Cell culture and reagents**

The human ovarian cancer cell lines, SKOV3 and OVCA3, were obtained from the American Type Culture Collection (Manassas, VA, USA). SNU-840 cells were purchased from the Korea Cell Line Bank (Seoul, South Korea). RMG-I cells were purchased from the Japanese Collection of Research Bioresources Cell Bank (Tokyo, Japan). OVCA433 and OVCA429 cells were obtained from the Catholic University of Korea, St. Vincent’s Hospital. Immortalised HOSE8695 (IHOSE8695) cells were established previously [1]. All cell lines were cultured in Dulbecco’s modified Eagle’s medium supplemented with 10% fetal bovine serum (HyClone, Logan, UT, USA) and 1% antibiotics (Cellgro, Manassas, VA, USA) and incubated at 37 °C in a humidified incubator with 5% CO_2_. Poly (2-hydroxyethyl methacrylate) (poly-HEMA) was purchased from Sigma (St. Louis, MO, USA). HA130 was purchased from Tocris Bioscience/Bio-Techne (Bristol, UK). Oregon Green® 488-conjugated gelatin and Alexa Fluor™ 594 Phalloidin were purchased from Invitrogen (Carlsbad, CA, USA).

**Patient samples**

To measure ATX expression in clinical samples using ELISA, ascites and serum samples were obtained from patients with ovarian cancer, patients with benign tumours, and healthy individuals at the Gangnam Severance Hospital and the Korea Gynecologic Cancer Bank under the Bio & Medical Technology Development Program of the Ministry of the National Research Foundation (NRF), which is funded by the Korean government (MSIT) (NRF-2017M3A9B8069610). The study was approved by the Regional Institutional Review Board at the Gangnam Severance Hospital (IRB No. 3-2017-0045, Seoul, South Korea), and informed consent was obtained from all participants. The inclusion and exclusion criteria for this retrospective study are described in Table S5. This study did not require randomization and blinding because of its retrospective nature. However, after updating the clinical information, the names and numbers of the patients were anonymised and then statistically analysed. The detailed patient characteristics are listed in Supplementary Table S1. Ovarian cancer was staged according to the International Federation of Gynecology and Obstetrics (FIGO) classification and graded according to the World Health Organization grading system. Clinical and pathological records were reviewed to collect data, including age, surgical procedure, survival time, survival status, tumour grade, and cell type.

**Determination of ATX level using ELISA**

The levels of ATX in the serum or ascites were determined using the Human ENPP-2/Autotaxin Quantikine ELISA Kit (R&D Systems, Minneapolis, MN, USA) according to the manufacturer’s instructions. All samples were examined in duplicate, and the mean values were used for statistical analysis. The human material used in this study was randomly selected. ATX clinical value was analysed by classifying according to sample clinical information.

**Spheroid-forming assay**

Multicellular tumour spheroids were generated as previously described [2]. Briefly, the cancer cell suspensions were seeded in 6-well culture plates precoated with 1.2 mg/ml poly-HEMA solution. The cells were then allowed to grow for 10 min to 48 h and then stained with calcein-AM (Invitrogen) to visualise the cells within the spheroid. Randomly selected fields (10×) from each well were imaged using a fluorescence microscope (EVOS FL; AMG), and the area of the spheroid was calculated using the Image J software.

**Real-time PCR**

Total RNA (1.25 µg), extracted from cancer cells using the TRIzol reagent (Invitrogen), was used for cDNA synthesis with the Maxima first-strand cDNA synthesis kit (Thermo Fisher Scientific, Villebon-sur-Yvette, France) according to the manufacturer’s protocol. Real-time PCR was conducted using the TOPreal qPCR 2X premix (Enzynomics, Daejeon, Republic of Korea) and Applied Biosystems 7300 real-time PCR system (Applied Biosystems, Darmstadt, Germany) with the primers listed in Supplementary Table S5. The reaction conditions were as follows: preincubation at 94 °C for 10 min, followed by 40 cycles at 94 °C for 10 s, 60 °C for 30 s, and 72 °C for 15 s and a melting curve procedure during which the temperature was increased from 60 °C to 95 °C. Relative mRNA expression levels were calculated using the comparative cycle threshold (2^−ΔΔ^Ct) method, with the 18S rRNA gene as an endogenous control to normalise the data.

**Western blot analysis**

Cancer cells were harvested and lysed with ice-cold RIPA buffer (25 mM tris–HCl, pH 7.6, 150 mM NaCl, 1% NP-40, 1% sodium deoxycholate, and 0.1% sodium dodecyl sulfate) containing a protease inhibitor cocktail (Roche Applied Science, Mannheim, Germany). Proteins were resolved using 10% sodium dodecyl sulphate-polyacrylamide gel electrophoresis and then transferred onto a nitrocellulose membrane (Pierce, Rockford, IL, USA) using an electric transfer system. The membrane was incubated with the following antibodies: anti-ATX (#sc-374222; dilution 1:1000) obtained from Santa Cruz Biotechnology (Santa Cruz, CA, USA), anti-integrin β1 (#9699), anti-integrin β3 (#13166), anti-integrin β4 (#14803), anti-integrin β5 (#3629), anti-integrin αV (#4711), and anti-integrin α5 (#4705) all in dilution 1:1000 obtained from Cell Signaling Technology, Inc. (Danvers, MA, USA). Anti-β-actin (Sigma–Aldrich) was used as the loading control. The membrane was then incubated with horseradish peroxidase-conjugated anti-mouse (#7076) or anti-rabbit (#7074) [all in dilution 1:1000 obtained from Cell Signaling Technology, Inc.] secondary antibodies, and the immunoreactive bands were visualised using enhanced chemiluminescence reagents (Santa Cruz Biotechnology).

**Small interfering RNA (siRNA) transfection**

The specific siRNAs against *ATX, SOX2*, and integrin β1 and the negative control siRNA were purchased from Santa Cruz Biotechnology, Inc. Cells were seeded at 2 × 10^5^ cells per 6-well plates and transfected with siRNA (10 nM) using the Lipofectamine RNAiMAX reagent (Invitrogen) according to the manufacturer’s instructions.

**Autotaxin expression constructs and transfection**

Plasmids containing the coding region of human autotaxin (PCMV6-ENPP2-Myc-DDK-tagged; Cat.RC200003, Origene) or the empty vector (PCMV6; Cat.PS100001, Origene) were transfected into cells using Lipofectamine 2000 reagent (Invitrogen) according to the manufacturer’s recommendations. Whole-cell lysates were then subjected to western blotting analysis to confirm autotaxin expression.

**Isolation of cell-free primary spheroid ovarian cancer-derived ascitic fluid for prospective study**

Primary ovarian cancer-derived, cell-free ascitic fluid was isolated from patients with ovarian cancer or benign tumours at the Gangnam Severance Hospital. The study was approved by the Regional Institutional Review Board at the Gangnam Severance Hospital (IRB No. 3-2021-0231, Seoul, South Korea), and informed consent was obtained from all participants. Ascitic fluid obtained from patients with ovarian cancer or benign tumours was placed in sterile tubes and centrifuged at 652 × *g* for 20 min at 4 °C. The cell-free supernatants were stored at −80 °C until assayed. To obtain a spheroid from which fibroblasts could be removed, the ovarian cancer-derived ascitic fluid was placed in a culture dish for 30 min [30,31], and the supernatant was centrifuged at 652 × *g* for 10 min. Thereafter, for RBC removal, pellets containing the spheroid cancer cells were treated with the RBC lysis buffer and stored at −80 °C with STEM-CELLBANKER GMP Grade (ZENOGEN PHARMA Co., Fukushima, Japan) until further investigation.

**Invadopodia assay**

To study the activity of invadopodia, coverslips were coated with Oregon Green 488-conjugated gelatin (Invitrogen) and incubated in a 12-well plate at 37 °C in a CO_2_ incubator for 2 h. The cells were seeded onto the Oregon Green 488-conjugated gelatin-coated coverslips in a complete medium and incubated for 16 h for gelatin degradation before washing with phosphate-buffered saline (PBS) and fixing with 4% paraformaldehyde. The cells were stained with Alexa Fluor™ 594 Phalloidin and DAPI (4′,6-diamidino-2-phenylindole) to observe F-actin distribution and the nucleus, respectively, before visualisation with a confocal microscope. To quantify the gelatin degradation activity of invadopodia, 10 randomly selected fields on the coverslips were imaged with a 60× objective lens prior to quantification. The experiments were repeated at least three times. The degraded area was quantified using the ImageJ software, and intensity profiles of the fluorescence signals along the lines indicated were analysed with the ZEN 3.3 software.

**Co-Immunoprecipitation (co-IP)**

Cells were harvested and lysed with ice-cold lysis buffer [20 mM Tris-HCl pH 8.0, 137 mM NaCl, 1% Nonidet P-40(NP-40), 2 mM EDTA] for 30 min. Cell lysates were incubated with anti-integrin β1 antibody (#sc-374429; dilution 1:100; Santa Cruz Biotechnology) overnight at 4 °C and mixed with Protein G-Agarose (Life Technologies) for 2 h. Beads were washed five times with ice-cold lysis buffer. Bound proteins were eluted by boiling in Laemmli sample buffer for 5 min. Samples were subjected to immunoblotting with anti-ATX antibody (#sc-374222; dilution 1:1000; Santa Cruz Biotechnology).

**Adhesion assays**

Cells were pre-stained with calcein-AM (Invitrogen), and resuspended cells at a density of approximately 1 × 10^5^ cells/ml were added to each well of a 12-well plate. The cells were then allowed to adhere to the bottom of the wells for 15 or 60 min in a CO_2_ incubator at 37 °C. Subsequently, the unbound cells were removed by aspiration and washed with PBS. The adherent cells were counted, and randomly chosen fields (4×) in each well were imaged using a fluorescence microscope (EVOS FL; AMG).

**Statistical analysis**

Most statistical analyses were performed with the Student’s t-test, Mann–Whitney test, or Spearman’s rank correlation coefficient using GraphPad Prism 7 (GraphPad Software, Inc., La Jolla, CA, USA). Multivariate analysis was performed using SPSS for Windows, version 26.0 (IBM Corp., Armonk, NY, USA). Receiver operating characteristic (ROC) curve analysis was performed using MedCalc statistical software version 20.019 (MedCalc Software Ltd., Ostend, Belgium). A chi-square test was performed to test the differences between categorical variables. Results are expressed as mean ± standard deviation. The median is represented using a horizontal bar in the centre of the box plot. Most significant differences were determined by an unpaired two-sided Student’s *t*-test or Mann–Whitney test. The Mann-Whitney-U test was used to compare two independent nonparametric samples. Statistically significant differences between grade I and grade II/II in ascites or serum from patients with ovarian cancer were determined using an unpaired, one-sided Mann–Whitney test. *P*-values < 0.05 were considered significant.

**Additional information**

**Ethics approval and consent to participate:** All clinical samples were approved by the Regional Institutional Review Board at the Gangnam Severance Hospital (IRB No. 3-2017-0045, IRB No. 3-2021-0231, Seoul, South Korea), and informed consent was obtained from all participants. The study was performed in accordance with the Declaration of Helsinki.

**Data availability:** The data generated in this study are publicly available in Oncomine ([www.oncomine.org](http://www.oncomine.org)) at TCGA_Ovarian Cancer and in cBioPortal for Cancer Genomics at TCGA PanCancer Atlas Studies. The data generated in this study are available within the article and its supplementary data files.

**References**

1. Shin HY, Yang W, Lee EJ, Han GH, Cho H, Chay DB, Kim JH. Establishment of five immortalized human ovarian surface epithelial cell lines via SV40 T antigen or HPV E6/E7 expression. PLoS One. 2018;13:e0205297.

2. Shepherd TG, Thériault BL, Campbell EJ, Nachtigal MW. Primary culture of ovarian surface epithelial cells and ascites-derived ovarian cancer cells from patients. Nat Protoc 2006;1: 2643-9.


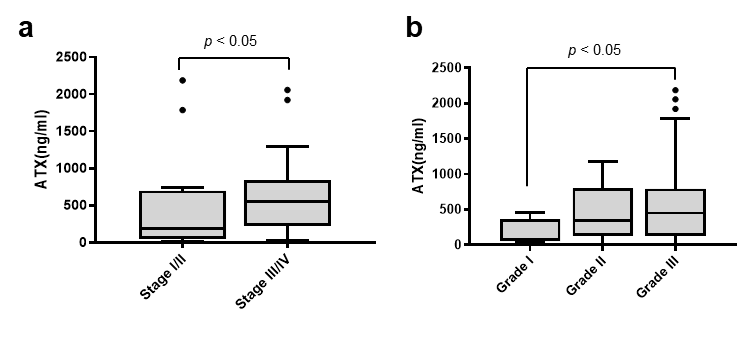


**Supplementary Figure S1. Elevated ascitic ATX levels in high-grade serous EOC.**

(a–b) Box and whisker plots showing ascitic ATX levels in patients with serous tumors at different stages (a), and grades (b). The one-tailed Mann–Whitney U test was used to evaluate statistical significance.


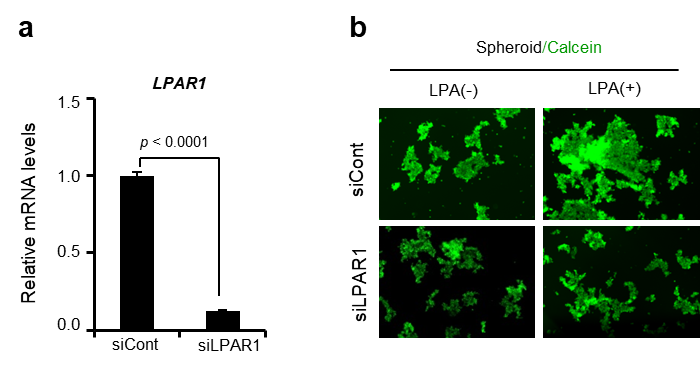


**Supplementary Figure S2. Effect of LPAR1 loss in LPA-induced spheroid formation in SKOV3 cells.**

(a) SKOV3 cells were transfected with LPAR1 siRNAs or siCont. Cell lysates were subjected to real-time PCR with the specific primers for *LPAR1*. The 18S rRNA gene was used as the endogenous control to normalise the data. Data are expressed as the means ± SD. (b) Effect of *LPAR1* loss on spheroid formation in SKOV3 cells. The cells were transfected with *LPAR1* siRNA or siControl. After 24 h, the cells were plated in poly-HEMA-coated dishes and incubated for 24 h in the presence or absence of LPA (1 μm). Spheroid cells were stained with calcein and imaged using an EVOS microscope. Scale bar = 100 μm.

**
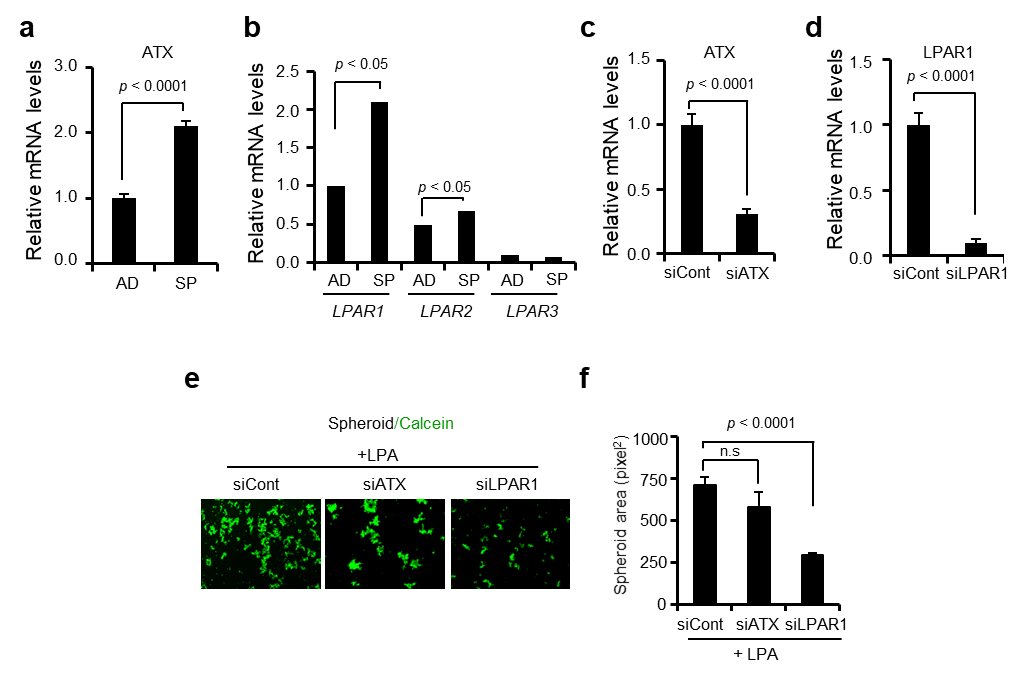
**

**Supplementary Figure S3. Effect of LPAR1 loss in LPA-induced spheroid formation in OVCAR429 cells.**

(a–b) Increased expression of *ATX* (a) and *LPAR* (b) genes in 3D spheroid culture of OVCAR429 cells determined using real-time PCR. Cells were plated onto poly-HEMA-coated dishes for 24 h. The cells were subjected to real-time PCR with the specific primers for *ATX* (a) and *LPARs* (b; *LPAR1, LPAR2,* and *LPAR3*). The 18S rRNA gene was used as the endogenous control to normalise the data. (c–d) Real-time PCR was performed with specific primers against *ATX* (c) and *LPAR1* (d). Note significant knockdown of ATX and LPAR1 expression after transfection of OVCAR429 cells with ATX siRNA and LPAR1 siRNA, respectively. Non-targeting siRNA (siCont) was used as a control. The 18S rRNA gene was used as the endogenous control to normalise the data. (e–f) Effect of ATX or LPAR1 depletion on spheroid formation in OVCAR429 cells. Cells were transfected with siRNA specific to the *ATX* or *LPAR1* genes. After 24 h, the cells were plated in poly-HEMA-coated dishes in the presence or absence of LPA (1 μΜ) for 24 h. Then, cells were stained with calcein and imaged using an EVOS microscope (e). Spheroid areas were calculated using the ImageJ software (f).

**
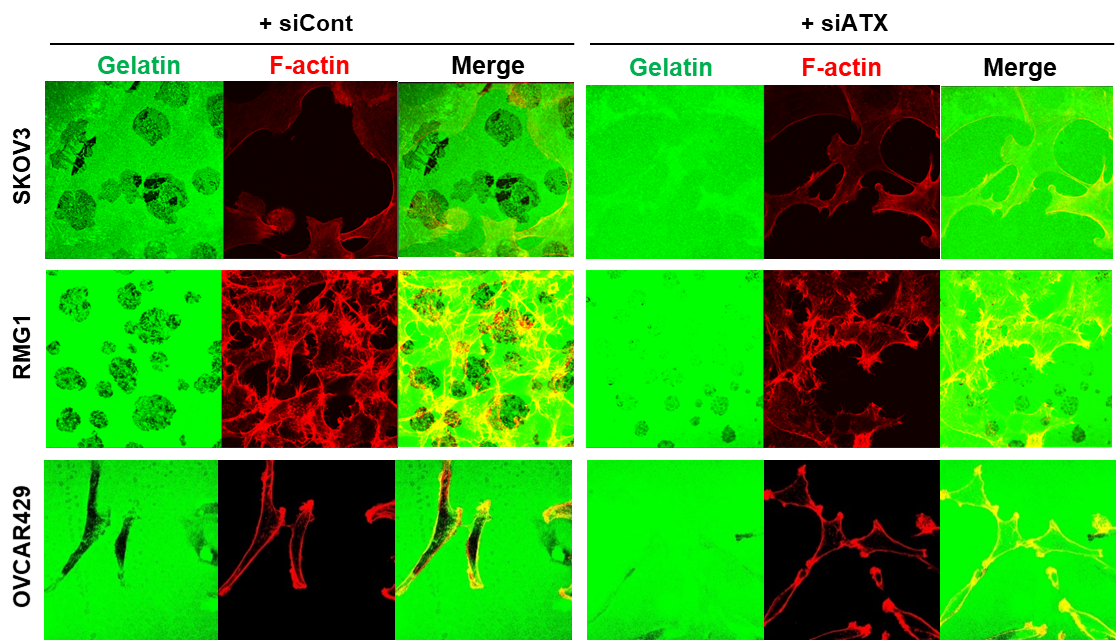
**

**Supplementary Figure S4. Effect of ATX-deficiency on invadopodia formation in ovarian cancer cells.**

Impairment of invadopodia formation by ATX silencing in SKOV3, RMG1, and OVCAR429 cells. Cells transfected with ATX siRNA were plated onto dishes coated with FITC-labeled gelatin. After 16 h, the cells were fixed with 4% paraformaldehyde and stained with Alexa Fluor 594-Phalloidin. The area of degraded gelatin was visualised using a confocal microscope. Representative images from each experimental group are shown.

**
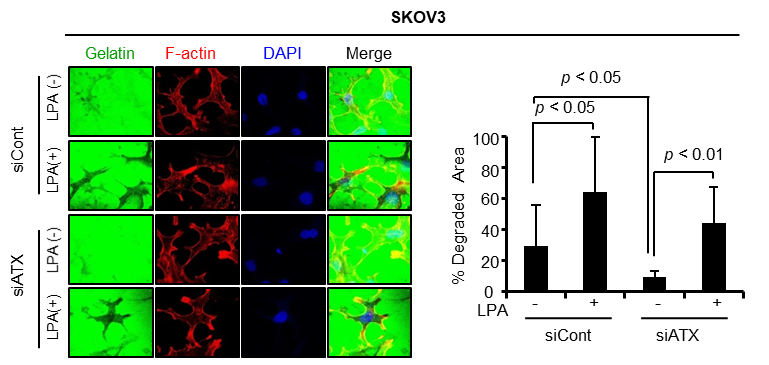
**

**Supplementary Figure S5. Effect of LPAR1-deficiency on LPA-stimulated invadopodia formation in ovarian cancer cells.**

Impairment of invadopodia formation by LPAR1 silencing in SKOV3 cells. Cells transfected with LPAR1 siRNA were plated onto dishes coated with FITC-labeled gelatin in the presence or absence of LPA (1 μΜ). After 16 h, the cells were fixed with 4% paraformaldehyde and stained with Alexa Fluor 594-Phalloidin and DAPI. The area of degraded gelatin was visualised using a confocal microscope. Representative images from each experimental group are shown (*left*). Gelatin degraded area was calculated using ImageJ (r*ight*).

**
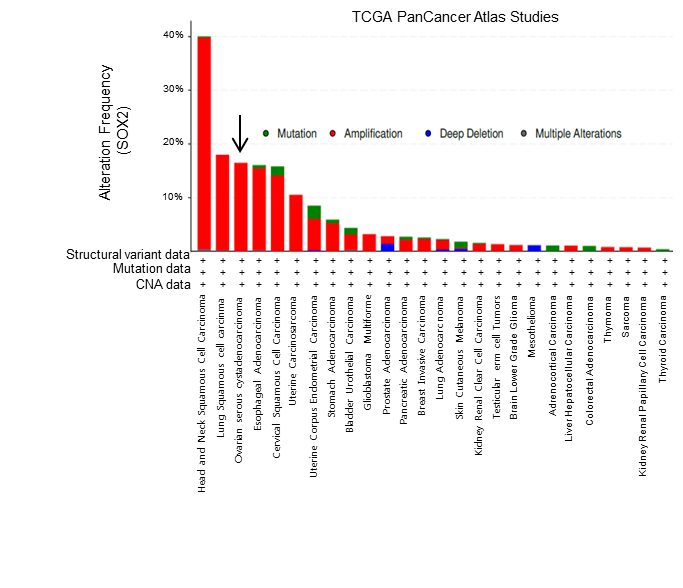
**

**Supplementary Figure S6.** The gene alteration frequency with mutation type and mutation sites of *SOX2* in TCGA tumours were analysed using the cBioPortal tool.

**
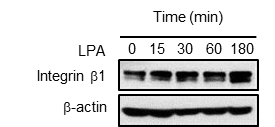
**

**Supplementary Figure S7. Immunoblotting analysis of integrin β1 expression after LPA stimulation in OVCAR429 cells.**

Cells were treated with LPA (1 μM) for 15, 30, 60, and 180 min. Cell lysates were subjected to immunoblotting with an anti-integrin β1 antibody. Anti-β-actin was used as the loading control.

**
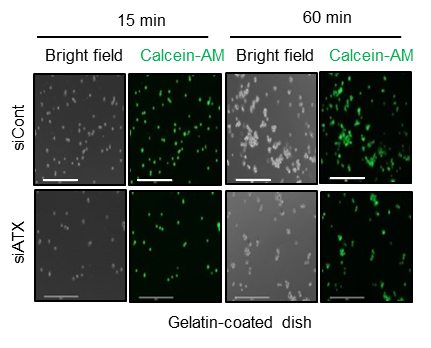
**

**Supplementary Figure S8. ATX deficiency impairs the adhesion ability of ovarian cancer cells.**

SKOV3 cells were stained with calcein-AM (10 μΜ) for 5 min and then seeded at a density of 4 × 10^5^ in six wells. After incubating for 15 and 60 min, the non-adherent cells were removed. Adherent cells were visualised under a fluorescence microscope (EVOS FL; AMG). Randomly selected fields (4×) in each well were imaged.

**
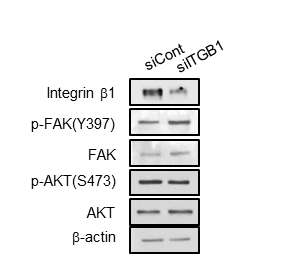
**

**Supplementary Figure S9. Effect of impaired integrin β1 expression on FAK and AKT signalling in SKOV3 cells.**

SKOV3 cells were transfected with siRNA integrin β1 or siRNA control. Cell lysates were subjected to immunoblotting with the indicated antibody. Anti-β-actin was used as the loading control.

**
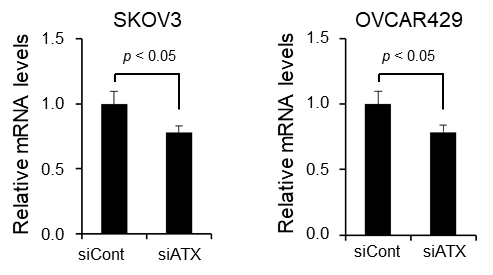
**

**Supplementary Figure S10. Impaired expression of USP10 in ovarian cancer cells.**

SKOV3 (*left*) and OVCAR429 (*right*) cells were transfected with ATX siRNA. Cell lysates were subjected to real-time PCR with the specific primers for USP10. The 18S rRNA gene was used as the endogenous control to normalise the data.

**
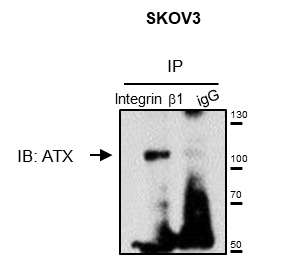
**

**Supplementary Figure S11. Co-immunoprecipitation of ATX and integrin β1 in SKOV3 cells.**

Cell lysates were immunoprecipitated using anti-integrin β1 antibody and immunoblotted with an anti-ATX antibody.

**Supplementary Table S1.** Association of ATX dichotomisation with clinical-pathological characteristics of patients with EOC

| Characteristics | Ascites | | | Serum | | |
| --- | --- | --- | --- | --- | --- | --- |
|  | ATX^low^,  *n* (%) | ATX^high^ *,*  *n* (%) | *p-*value | ATX^low^, *n* (%) | ATX^high^ *,*  *n* (%) | *p-* value |
| ***Diagnosis*** |  |  | 0.259 |  |  | 0.052 |
| Healthy | N.A | N.A |  | 0 (0.0) | 40 (35.7) |  |
| Benign | 11 (21.2) | 16 (14.2) |  | N.A | N.A |  |
| Cancer | 41 (78.8) | 97 (85.8) |  | 7 (100) | 72 (64.3) |  |
| ***Age*** |  |  | 0.475 |  |  | 0.755 |
| ≥50 | 30 (73.2) | 65 (67.0) |  | 2 (28.6) | 23 (31.9) |  |
| 50< | 11 (26.8) | 32 (33.0) |  | 5 (71.4) | 49 (68.1) |  |
| ***FIGO stage*** |  |  | 0.009* |  |  | 0.534 |
| I | 16 (39.0) | 15 (15.5) |  | 1 (14.3) | 23 (31.9) |  |
| II | 3 (7.3) | 5 (5.2) |  | 1 (14.3) | 4 (5.6) |  |
| III | 11 (26.8) | 46 (47.4) |  | 2 (28.6) | 30 (41.7) |  |
| IV | 1 (2.4) | 12 (12.4) |  | 1 (14.3) | 4 (5.6) |  |
| Recurrence | 10 (24.4) | 19 (19.6) |  | 2 (28.6) | 11 (15.3) |  |
| ***Differentiation*** |  |  | 0.130 |  |  | 0.707 |
| Grade 1 | 7 (17.1) | 6 (6.2) |  | 1 (14.3) | 5 (6.9) |  |
| Grade 2 | 5 (12.2) | 14 (14.4) |  | 5(71.4) | 60 (83.3) |  |
| Grade 3 | 27 (65.9) | 64 (66) |  | 0 (0.0) | 0 (0.0) |  |
| Unclassified | 2 (4.9) | 13 (13.4) |  | 1 (14.3) | 7 (9.7) |  |
| ***Histology*** |  |  | 0.003* |  |  | 0.341 |
| Serous | 25 (61.0) | 69 (71.1) |  | 7 (100) | 48 (66.7) |  |
| Mucinous | 0 (0.0) | 13 (13.4) |  | 0 (0.0) | 7 (9.7) |  |
| Endometrioid | 5 (12.2) | 3 (3.1) |  | 0 (0.0) | 7 (9.7) |  |
| Clear | 11 (26.8) | 12 (12.4) |  | 0 (0.0) | 10 (13.9) |  |
| ***CA125 (U/mL)*** |  |  | 0.025* |  |  | 0.856 |
| ≥35 | 32 (78.0) | 89 (91.8) |  | 6 (85.7) | 60 (83.3) |  |
| 35< | 9 (22.0) | 8 (8.2 ) |  | 1 (14.3) | 9 (12.5) |  |
| Unclassified | 0 (0.0) | 0 (0.0) |  | 0 (0.0) | 3 (4.2) |  |

EOC, epithelial ovarian cancer; *n* = Number of patients; N/A, not applicable; FIGO, Federation of Gynecology and Obstetrics. **p* < 0.05

**Supplementary Table S2.** Association of ATX dichotomisation with clinical-pathological characteristics of patients with HGSOC.

| Clinical characteristics | Ascites | | |
| --- | --- | --- | --- |
|  | ATX^low^, *n* = 20 (%) | ATX^high^ *, n* = 61(%) | *p-value* |
| *Age* |  |  | 0.258 |
| ≥ 50 | 18 (90.0) | 48 (78.7) |  |
| 50 < | 2 (10.0) | 13 (21.3) |  |
| *FIGO stage* |  |  | 0.007* |
| I | 4 (20.0) | 3 (4.9) |  |
| II | 2 (10.0) | 2 (3.3) |  |
| III | 5 (25.0) | 38 (62.3) |  |
| IV | 1 (5.0) | 8 (13.1) |  |
| Recurrence | 8 (40.0) | 10 (16.4) |  |
| *Differentiation* |  |  | 0.975 |
| Grade 1 | 0 (0) | 0 (0) |  |
| Grade 2 | 4 (20.0) | 12 (19.7) |  |
| Grade 3 | 16 (80.0) | 49 (80.3) |  |
| *CA125 (U/mL)* |  |  | 0.610 |
| ≥ 35 | 18 (90.0) | 57 (93.4) |  |
| 35 < | 2 (10.0) | 4 (6.6) |  |

HGSOC, high-grade serous ovarian carcinoma; *n* = Number of patients; N/A, not applicable; FIGO, Federation of Gynecology and Obstetrics. **p* < 0.05

**Supplementary Table S3. ROC curve analysis for ascitic ATX combined with serum CA125 in patients with ovarian cancer.**

| **Group** | **Biomarkers** | **ROC-AUC [95% CI]** | **Sensitivity**  **[95% CI]** | **Specificity**  **[95% CI]** | **PPV (%)**  **[95% CI]** | **NPV (%)**  **[95% CI]** |
| --- | --- | --- | --- | --- | --- | --- |
| ***Whole*** | **Ascitic ATX** | 0.709  [0.633–0.777] | 52.90  [44.2–61.4] | 88.89  [70.8–97.6] | 96.1  [89.2–98.6] | 27.0  [22.8–31.5] |
|  | **Serum CA125** | 0.771  [0.699–0.834] | 87.59  [80.9–92.6] | 66.67  [44.7–84.4] | 93.7  [89.5–96.4] | 48.5  [35.7–61.5] |
|  | **Combined ascitic ATX and serum CA125** | 0.842  [0.776–0.894 | 87.59  [80.9-92.6] | 66.67  [44.7–84.4] | 93.7  [89.5–96.4] | 48.5  [35.7–61.5] |

PPV, positive predictive value; NPV, negative predictive value.

**Supplementary Table S4. Inclusion and exclusion criteria for the retrospective study.**

| Inclusion criteria | 1. Patients diagnosed with endometrial cancer or benign tumour from January 2005 to November 2021. 2. Patients ≥ 19 years old. 3. Participants who agreed to provide tissues to the human material bank during surgery and biopsy to be performed for treatment and diagnosis. |
| --- | --- |
| Exclusion criteria | 1. History of infections or other serious medical problems that impair the patient’s functioning and make protocol compliance difficult. 2. Patient who did not seem like they will be able to comply with the clinical trial procedures and requirements, as judged by the investigator. |

**Supplementary Table S5. Sequence of the primers used for real-time PCR.**

| **Gene** | **Accession No.** | **Sequence (5′ to 3′)** | **Size (bp)** |
| --- | --- | --- | --- |
| *SOX2* | NM_003106.3 | F, 5′- ACCAGCTCGCAGACCTACAT-3′  R, 5′- TGGAGTGGGAGGAAGAGGTA-3′ | 154 |
| *OCT4* | NM_001285987.1 | F, 5′- CTGCAGTGTGGGTTTCGGGCA-3′  R, 5′- CTTGCTGCAGAAGTGGGTGGAGGAA-3′ | 169 |
| *ALDH* | NM_000689.4 | F, 5′- TACTCACCGATTTGAAGATT-3′  R, 5′- TTGTCAACATCCTCCTTATC-3′ | 151 |
| *LPAR1* | NM_001401.5 | F, 5′-TCCTGTCCCGCGCCAGGTACAC-3′  R, 5′-GGTGGTGAACACGCCCCAGAACT-3′ | 101 |
| *LPAR2* | NM_001395660.1 | F, 5′-ACCGCAGTGTGATGGCCGTG-3′  R, 5′-TAGGAGCGGCTGAGCAGGGG-3′ | 178 |
| *LPAR3* | NM_012152.3 | F, 5′-GCCGTGGAGAGGCACATGTC-3′  R, 5′-TGGCGATGGCCCAGACAAGC-3′ | 100 |
| *USP10* | NM_001272075.2 | F, 5′-GATCCTCTGAAACCGGAACA-3′  R, 5′-AGAGTGCATCACCTCCTGCT-3′ | 219 |
| *18S* | NM_022551.3 | F, 5′- TCCAGGTCTTCACGGAGCTTGTT-3′  R, 5′- GGATGTAAAGGATGGAAAATACA-3′ | 210 |
